# Chemical Compounds, Pharmacological and Toxicological Activity of *Brugmansia suaveolens*: A Review

**DOI:** 10.3390/plants9091161

**Published:** 2020-09-08

**Authors:** Vera L. Petricevich, David Osvaldo Salinas-Sánchez, Dante Avilés-Montes, Cesar Sotelo-Leyva, Rodolfo Abarca-Vargas

**Affiliations:** 1 Faculty of Medicine, Autonomous University of the State of Morelos (UAEM), Street: Leñeros, esquina Iztaccíhuatl s/n. Col. Volcanes, Cuernavaca 62350, Morelos, Mexico; vera.petricevich@uaem.mx; 2 Biodiversity and Conservation Research Center, Autonomous University of the State of Morelos (UAEM), Av. Universidad 1001, Col. Chamilpa, Cuernavaca 62209, Morelos, Mexico; davidos@uaem.mx; 3 Faculty of Biological Science, Autonomous University of the State of Morelos (UAEM), Av. Universidad 1001, Col. Chamilpa, Cuernavaca 62209, Morelos, Mexico; dante.aviles@uaem.mx; 4 Faculty of Chemistry-Biological Sciences, Autonomous University of Guerrero Av. Lázaro Cárdenas s/n, South University City, Chilpancingo 39000, Guerrero, Mexico; cesarsotelo@uagro.mx

**Keywords:** alkaloids, terpenoid, scopolamine, antinociceptive, nematicide, toxicity

## Abstract

This study investigates updated information in different search engines on the distribution, phytochemistry, pharmacology, and toxicology of *Brugmansia suaveolens* (Solanaceae) using the extracts or chemical compounds at present. This plant has been used in traditional medicine in different cultures as a hallucinatory, analgesic, aphrodisiac, nematicide, sleep inducer, and muscle relaxant, as well as a treatment for rheumatism, asthma, and inflammation. The flowers, fruits, stems, and roots of the plant are used, and different chemical compounds have been identified, such as alkaloids, volatile compounds (mainly terpenes), coumarins, flavonoids, steroids, and hydrocarbons. The concentration of the different compounds varies according to the biotic and abiotic factors to which the plant is exposed. The toxic effect of the plant is mainly attributed to atropine and scopolamine, their averages in the flowers are 0.79 ± 0.03 and 0.72 ± 0.05 mg/g of dry plant, respectively. Pharmacological studies have shown that an aqueous extract exhibits the antinociceptive effect, at doses of 100 and 300 mg/kg i.p. in mice. On the other hand, the ethanolic extract at 1000 mg/L, showed a nematocidal activity in vitro of 64% against *Meloidogyne incognita* in 72 h. Likewise, it showed a 100% larvicidal activity at 12.5 mg/L against *Ancylostoma spp*. In another study, the lethal activity of shrimp in brine from an ethanolic extract showed an LC_50_ of 106 µg/mL at double serial concentrations of 1000–0 (µg/mL). Although there are pharmacological and phytochemical studies in the plant, they are still scarce, which has potential for the examination of the biological activity of the more than one hundred compounds that have been reported, many of which have not been evaluated.

## 1. Introduction

*Brugmansia suaveolens* (Humb. and Bonpl. ex Willd.) Bercht. and J.Presl is widely distributed in the world both as a spontaneous species and as an ornamental plant [1], mainly in areas with climates ranging from tropical and subtropical to temperate [2]. It belongs to the Solanaceae family, and according to *The Plant List*, 12 are recognized in this genus, including hybrids and a subspecies (*Brugmansia arborea*, *B*. × *candida*, *B*. × *cubensis*, *B*. × *dolichocarpa*, *B*. × *insignis*, *B*. *longifolia*, *B*. *pittieri*, *B*. × *rubella*, *B*. *sanguinea*, *B*. *sanguinea* subsp. *vulcanicola*, *B*. *suaveolens,* and *B*. *versicolor*) [1]. The objective of this review is to present complete and updated information on the current research regarding the distribution, phytochemistry, pharmacology, and toxicology of *B*. *suaveolens*, in order to identify its therapeutic potential and open new research opportunities. The most salient data were searched using the keyword “*Brugmansia suaveolens*” in Google Scholar, ScienceDirect, Wiley, Taylor and Francis, and PubMed. The synonyms, according to *The Plant List,* of *B*. *suaveolens* (Humb. and Bonpl. ex Willd.) Bercht. and C. Presl, are *Brugmansia albidoflava* (Lem.) Verschaff. ex Bosse (unresolved), *Datura albidoflava* Lem. (synonym), *Datura arborea* Mart. (synonym), *Datura gardneri* Hook. (synonym), *Datura suaveolens* Humb. and Bonpl. ex Willd. (synonym), *Datura suaveolens* f. *albidoflava* (Lem.) Voss (synonym), *Datura suaveolens* var. *macrocalyx* Sendtn. (synonym), *Pseudodatura suaveolens* Zijp (unresolved), and *Stramonium arboreum* Moench (unresolved) [1].

*Brugmansia suaveolens* is known by various names in different areas of the world, such as in Mexico (Floripondio or florifundio) [3], (Toloache de castilla) [4], Argentina (Floripón) [5], Peru (Misha Colambo (Snake)) [6], (Floripondio) [7], (Toe, Toe de flor blanca) [8], Brazil (Trombeteira or Cartucheira) [9], (Trompeta de Ángel) [10], Sweden (Angel’s Trumpet) [11], Sir Lanka (Attana) [12], and in Indonesia (Kecubung Bunga Kuning and Kecubung Bunga Putih) [13], (Cubung) [14], Pakistan (Shaitani ganti/Bel Boti) [15], and Butan (Gangmeto) [16]. In many other countries, *B*. *suaveolens* is better known by the name Angel’s Trumpet.

## 2. Botany

### 2.1. Taxonomical Classification

*Brugmansia suaveolens*, was discovered by Alexander von Humbold and Aimé Bonpland (Humb. Bonpl. Ex Willd.). It was first formally described by Friederich von Berchtold and Jan Presl, and published in Hortus suburbanus Londinensis [1] (Table 1).

**Figure 1:**
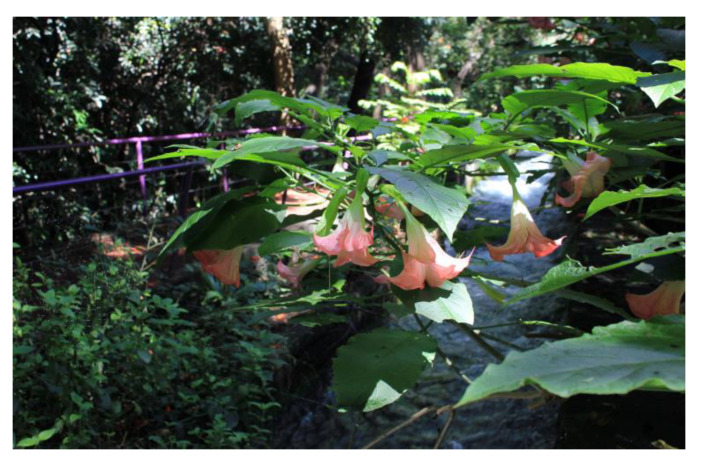
*Brugmansia suaveolens*.

*Brugmansia suaveolens* belongs to the group of woody plants [17], considered shrubs or small trees (Figure 1), with a length ranging from 1 to 6 m high. The petiole is 2–5 cm long; the leaf lamina, with the widest end below the middle part known as an elliptical 15–30 cm long and 5–12 cm wide; the corolla is formed by a tube with lobes of 25–30 cm long; and the basal half is a narrow tubular shape and abruptly expands to form extended lobes 10–15 cm long. The color of the flower is white or reddish. The fruit is narrow at the end and wide in the middle part, and is 20 cm long with a 2.5 cm diameter. Flowering begins in January and from April to November. It bears fruit from May to June and in October [3].

### 2.2. Distribution

**Figure 2:**
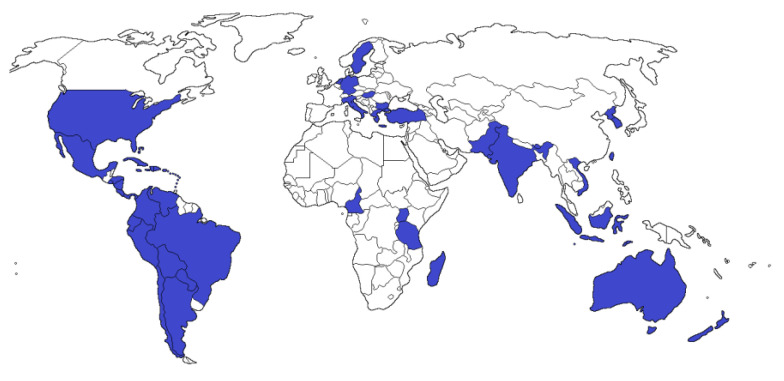
Global distribution map of *Brugmansia suaveolens*.

*Brugamnsia suaveolens* is widely distributed around the world (Figure 2), including in the USA [2], Mexico [18], Honduras, Nicaragua, Panama, El Salvador, Paraguay, and the Antilles [3], Argentina [19], Bolivia [20], Costa Rica [21], Colombia [22], Ecuador [23], Venezuela [24], Peru [6], Chile [25], Brazil [18], Korea [26], Vietnam [27], Taiwan [28], India [29], Indonesia [30], Butan [16], Sri Lanka [12], Pakistan [15], Turkey [31], Australia [32], New Zealand [33], Cameroon, Madagascar, Tanzania [3], Uganda [34], Italy, Bulgaria [35], Netherlands [35], Germany [36], Hungary [37], Greece [38], and Sweden [39].

### 2.3. Ethnobotany

Many of these species are mainly appreciated as ornamentals, because of their ease of cultivation and the production of their characteristic flower smell at dusk [3], reaching its maximum peak at 21:00 [22]. *B*. *suaveolens* is also used in traditional medicine [3], despite being documented in the literature that its greatest use is as a hallucinogenic in shamanic rituals in some populations of Latin America [6], among them being some ethnic groups from the Amazon of Peru and Ecuador [40]. In the Inga people of Colombia, it is used externally to ward off the evil spirits that cause insomnia [41]. However, the first instances of its medicinal use were the Spanish in colonial times, where these plants were used for the treatment of rheumatism, infections, and asthma [42].

It is used to calm toothache [3]; to treat inflammation from trauma [6]; reduce general body inflammation [14]; for sores; to heal wounds without scars [6]; for treating pain in general [9], especially chest pain [43]; to treat abscesses, dermatitis, and fungal infections of the skin [44]; for snake bites; as an aphrodisiac [45]; for diarrhea [15]; gonorrhea; and for loss of appetite [14]. The flower buds are used to treat eye pain [46] and coughing [4].

## 3. Phytochemistry

Chemical studies of this medicinal species date back to 1996 [47]. Such studies were the first qualitative on groups of compounds, where they were identified as amines, carbohydrates [48], alkaloids, phenolic compounds, flavonoids, steroids, terpenoids, tannins, anthraquinone glycosides, saponins, and triterpenes. The quantification of the alkaloids (5.903 ± 0.01333 mg/g), phenolic compounds (3.435 ± 0.0110 mg/g), and flavonoids (9.945 ± 0.0256 mg/g) was also carried out from the ethanolic extract of the flowers [49]. The concentrations of such compounds can vary, as in living flowers, they show a continuous change in the profile of their volatile compounds, which depend on intrinsic (genetic) and external factors (light, temperature, and water stress). In the case of the cut flowers, they suffer faster deterioration and a loss of volatile compounds [22]. Other factors that also affect it are attacks from pathogens (viruses, bacteria, fungi, and nematodes) and herbivores. The Marvin program was used to draw the structures of organic chemical compounds [50].

### 3.1. Alkaloids

**Figure 3:**
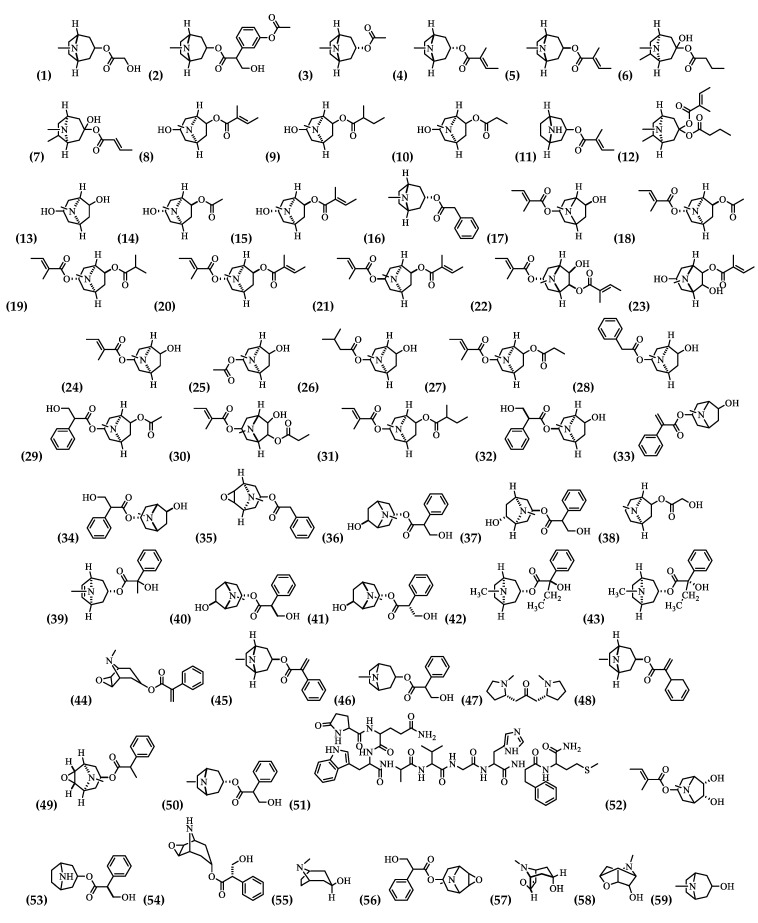
Structure of alkaloids from *Brugmansia suaveolens.*

Tropane alkaloids have anticancer activity [51]. Therefore, this group should be more studied in this regard. However, chemically, it is one of the most studied, where 59 alkaloids have been identified in the mature flowers, as well as in the immature flowers and fruits, corolla, flowers, roots, and flower nectar (Table 2 and Figure 3).

### 3.2. Volatile Compounds

**Figure 4:**
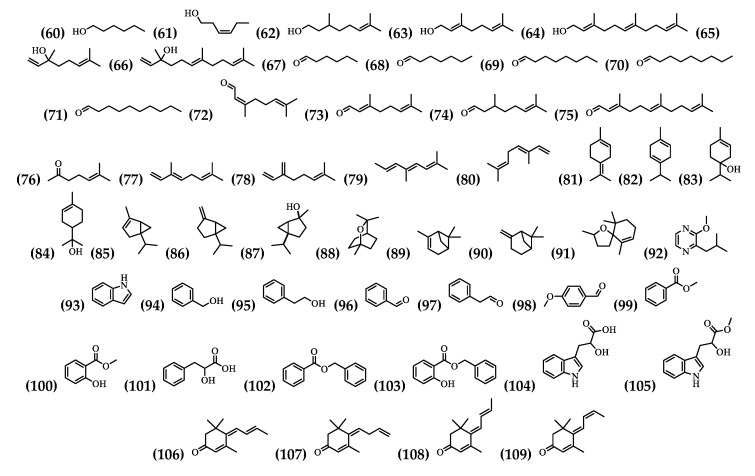
Structure of Volatile Compounds from *Brugmansia suaveolens*.

In the flowers and leaves, 50 volatile compounds have been identified and most of these compounds are found in the flowers (Table 3 and Figure 4).

### 3.3. Phenolic Compounds, Coumarin, and Flavonoids

**Figure 5:**
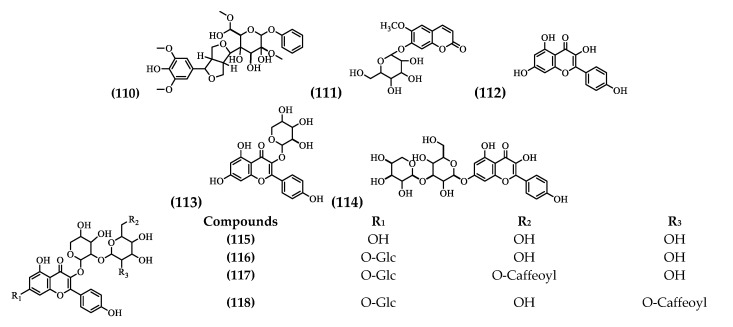
Structure of Phenolic Compounds, Coumarin, and Flavonoids from *Brugmansia suaveolens.*

A glycosylated phenolic compound, a coumarin, and seven flavonoids have been identified, in the flowers and leaves (Table 4 and Figure 5).

### 3.4. Steroids

**Figure 6:**
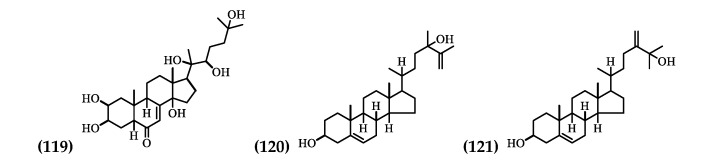
Structure of Steroids from *Brugmansia suaveolens.*

Three Steroids have been identified in the flowers and leaves (Table 5 and Figure 6).

### 3.5. Hydrocarbons

**Figure 7:**
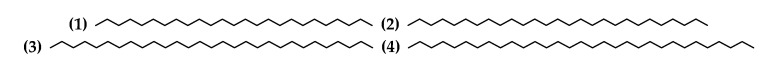
Structure of the hydrocarbons from *Brugmansia suaveolens.*

The presence of four hydrocarbons in *B*. *suaveolens* has been identified only in the flowers (Table 6 and Figure 7).

## 4. Pharmacological Activity

*Brugmansia suaveolens* is reported in traditional medicine in many Latin American countries; however, the first studies are from 22 years ago [4]. As there are very few pharmacological investigations of the plant, this is still an opportunity for future investigations.

### 4.1. Antinociceptive

The aqueous extract of *B*. *suaveolens* flowers was administered at doses of 100 and 300 mg/kg i.p. They significantly inhibited (*p* < 0.05) the induced contortions and increased the percentage of inhibition by acetic acid to 0.6% (3.0 ± 0.8 and 94.9%, and 0.6 ± 0.5 and 99%, respectively). Diclofenac 5 mg/kg i.p (43.4 ± 3.5 and 25.8%) was used as a positive control. An increase in the latency time was observed in the formalin test (20 µL of 2.5%); in the first phase (0–5 min), with a dose of 100 mg/kg (15.6 ± 4.2 s and 63.3%) and 300 mg/kg (0.3 ± 0.3 s and 98.6%) and diclofenac (43.6 ± 7.0 s and 0%), and the second phase (20–25 min) with a dose of 100 mg/kg (7.5 ± 2.8 s and 82.2%) and 300 mg/kg (0.0 ± 0.0 s and 100%) and diclofenac (7.0 ± 2.8 s and 69.6%) in male Swiss albino mice. An increase in the latency time was also observed in the hot plate and tail dip tests [9].

In another study of the aqueous extract of flowers of *B*. *suaveolens* on the probable antinociceptive mechanism of the 300 mg/kg dose, a mechanism on benzodiazepine receptors was found. Flumazenil (5 mg/kg, i.p.) was used as an antagonist [57].

### 4.2. Antimicrobial

The antibacterial activity of the aqueous extract of *B*. *suaveolens* flowers against *Bacillus thurigiensis* was evaluated in one study and showed no activity [58].

### 4.3. Nematicide

The ethanolic extract of flowers at a concentration of 1000 mg/L, showed a 64% in vitro nematocidal activity against *Meloidogyne incognita* within 72 h. [49]. In another study of the ethanolic extract of aerial parts (flowers, and flowers and stems), a 100% larvicidal activity at a dilution of 12.5 mg against *Ancylostoma* spp was shown [59].

### 4.4. Cytotoxicity

Studies of the cytotoxic evaluation of the aqueous extract of *B*. *suaveolens* were carried out in the Brine-shrimp model (*Artemia* sp., Artemiidae) during 24 h, the concentrations of 1000, 500, 250, 125, 62.5, 31.25, and 0 (µg/mL) were evaluated. An LC_50_ of 106 µg/mL was obtained [7].

### 4.5. Muscle Relaxer

*Brugmansia suaveolens* ethanol extract inhibits rabbit smooth muscle contractility at 100% at a concentration of 75.5 g/mL [4].

## 5. Toxicity

There are several factors (climatic and seasonal) that can increase or decrease the concentration of the alkaloids associated with the toxicity of the plant. It has been documented that it has been involved in poisoning in many parts of the world, and other species, such as *B*. *candida*, *B*. *sanguinea*, and *B*. × *candida*, are considered toxic in some places like Mexico, especially their seeds. It is documented that an intake of 4 to 5 g of raw leaf, or just a seed, can cause a child to die [3]. Among the most toxic compounds are atropine and scopolamine, and their averages in the flowers are 0.79 ± 0.03 and 0.72 ± 0.05 mg/g of dry plant, respectively; these concentrations will increase if the plant is fertilized with organic fertilizer (6 kg/m^2^ per year) [44]. In another study, a scopolamine concentration of 149.80 ± 6.01 µg/mL was determined in the flower nectar [37]. The plant parts that are the most involved in poisoning are flowers (77.5%), leaves (13.4%), fruits (4.5%), stem (2.3%), and root (2.3%) [60].

The signs and symptoms of Angel’s Trumpet poisoning are mydriasis, dry mouth, delirium, reddened skin [61], dry skin [60], agitation/aggressiveness, reduced bowel sounds [61], ileal paralysis, drowsiness [62], visual hallucinations, tachycardia, urinary retention, fever, increased systolic blood pressure, a Glasgow Coma Scale (GCS) of <12, [61], vertigo [60], decreased temperature and difficulty breathing prior to coma [3].

Unusual poisoning occurred in a five-year-old boy who consumed flowers, and as a consequence, unilateral tonic pupils and Guillain-Barré syndrome were observed [31].

## 6. Conclusions

This review details the distribution and ethnomedical, phytochemical, pharmacological, and toxicological uses of *B*. *suaveolens* in the world. The scientific investigations that have been carried out to date are scarce, the analgesic, cytotoxic, nematicidal, and antimicrobial activity has been studied. However, regarding it’s chemistry, it is important to highlight that 125 compounds have been reported and identified, and a high percentage are not associated with pharmacological activity. Ethnomedical uses reported around the world include its uses to treat pain, insomnia, rheumatism, infections, asthma, inflammation, sores, wounds, abscesses, dermatitis, snakebites, loss of appetite, coughs, and as an aphrodisiac. From this empirical and traditional knowledge in different countries, the scientific validation of this plant species emerges as a great area of opportunity, which provides an opportunity for interdisciplinary collaboration between different research groups.

## Figures and Tables

**Table 1:** Taxonomical classification of *Brugmansia suaveolens*.

Kingdom	Plantae
Subkingdom	Tracheobionta
Superdivision	Spermatophyta
Division	Magnoliophyta
Class	Magnoliopsida
Order	Solanales
Family	Solanaceae
Subfamilia	Solanoideae
Tribe	Datureae
Genus	*Brugmansia*
Species	*B. suaveolens* [1]

**Table 2:** Alkaloids from *Brugmansia suaveolens*.

No.	Compound Name	Parts Used
(1)	3-(Hydroxyacetoxy)-tropane	Roots [35]
(2)	3-(3′Acetoxytropoyloxy)-tropane	Flowers, Roots [35]
(3)	3α-Acetoxytropane	Root cultivation [36]
(4)	3α-Tigloyloxytropane	Flowers and Roots [35]
(5)	3β-Tigloyloxytropane	Flowers [35]
(6)	3-Hydroxy-6-methylbutyryloxy-tropane	Roots [35]
(7)	3-Hydroxy-6-methyl-butenoyl-oxytropane	Flowers [47]
(8)	3-Hydroxy-6-tigloyloxytropane	Root cultivation [36]
(9)	3-Hydroxy-6-(2-methyl butyryloxy)-tropane	Root cultivation [36], Flowers, Roots [35]
(10)	3-Hydroxy-6-propionyl-oxytropane	Flowers [47]
(11)	3-Tigloyloxynortropane	Flowers [35]
(12)	3-Tigloyloxy-6-methylbutyryloxytropane	Flowers, Roots [35]
(13)	3,6-Dihydroxytropane	Flowers [47]
(14)	3α-Hydroxy-6β-acetoxytropane	Flowers, Roots [35]
(15)	3α-Hydroxy-6β-tigloyloxytropane	Flowers, Roots [35]
(16)	3α-Phenylacetoxytropane	Roots [35]
(17)	3α-Tigloyloxy-6β-hydroxytropane	Flowers, Roots [35]
(18)	3α-Tigloyloxy-6β-acetoxytropane	Flowers [35]
(19)	3α-Tigloyloxy-6β-isobutyryloxytropane	Flowers, Roots [35]
(20)	3α,6β-Ditigloyloxytropane	Flowers, Roots [35] and Root cultivation [36]
(21)	3β,6β-Ditigloyloxytropane	Roots [35]
(22)	3α,6β-Ditigloyloxy-7β-hydroxytropane	Flowers [35]
(23)	3,6-dihydroxy-7-tigloyloxytropane	Flowers [35]
(24)	3-Tigloyloxy-6-hydroxytropane	Root cultivation [36]
(25)	3-Acetoxy-6-hydroxytropane	Root cultivation [36]
(26)	3-Isovaleryloxy-6-hydroxytropane	Roots [35]
(27)	3-Tigloyloxy-6-propionyloxytropane	Flowers, and Roots [35]
(28)	3-Phenylacetoxy-6-hydroxytropane	Flowers [35]
(29)	3-Tropoyloxy-6-acetoxytropane	Flowers [35]
(30)	3-Tigloyloxy-6-propionyloxy-7-hydroxytropane	Flowers, Roots [35]
(31)	3-Tigloyloxy-6-(2-methylbutyryloxy)-tropane	Root cultivation [36] and Roots [35]
(32)	7-Hydroxyhyoscyamine	Flowers [35]
(33)	6-Hydroxyapoatropine	Flowers [47], Root cultivation [36], Roots [35]
(34)	6-Hydroxyhyoscyamine	Flowers, Roots [35]
(35)	3-Phenylacetoxy-6,7-epoxytropane	Flowers [52]
(36)	6β-Hydroxyhyoscyamine	Root cultivation [36]
(37)	7β-Hydroxyhyoscyamine	Root cultivation [36]
(38)	6-Hydroxyacetoxytropane	Flowers, Roots [35]
(39)	6,7-Dehydronoratopine	Flowers [47]
(40)	6R-Hydroxyhyoscyamine	Flowers [47]
(41)	6S-Hydroxyhyoscyamine	Flowers [47]
(42)	6R-Hydroxynorhyoscyamine	Flowers [47]
(43)	6S-Hydroxynorhyoscyamine	Flowers [47]
(44)	Aposcopolamine	Flowers [47], Root cultivation [36], and Roots [35]
(45)	Apoatropine	Flowers [47], Root cultivation [36], and Roots [35]
(46)	Atropine	Root cultivation [36], and Corolla [53]
(47)	Cuscohygrine	Root cultivation [36]
(48)	Dihydroapoatropine	Flowers [47]
(49)	Dihydroaposcopolamine	Flowers [47]
(50)	Hyoscyamine	Root cultivation [36], Corolla [53], Roots [35], and Flowers [47]
(51)	Litorine	Flowers, and Roots [35]
(52)	Meteloidine	Flowers [35]
(53)	Norhyoscyamine	Flowers [47]
(54)	Norscopolamine	Flowers [47]
(55)	Pseudotropine	Root cultivation [36], Flowers, Roots [35]
(56)	Scopolamine	Ripe flowers, and immature flowers and fruits [54], Corolla [53], Flowers [47], Roots [35], and Flowers nectar [37]
(57)	Scopine	Root cultivation [36], Flowers [47]
(58)	Scopoline	Root cultivation [36]
(59)	Tropine	Flowers, and Roots [35]

**Table 3:** Volatile Compounds from *Brugmansia suaveolens*.

No.	Compound Name	Parts Used	No.	Compound Name	Parts Used
(60)	Hexanol	Flowers [22]	(85)	α-Tujene	Flowers [22]
(61)	(Z)-3-Hexen-1-ol	Flowers [22]	(86)	Sabinene	Flowers [22]
(62)	Citronellol	Flowers [22]	(87)	*trans*-Sabinene hydrate	Flowers [22]
(63)	Geraniol	Flowers [22]	(88)	1,8-cineol	Flowers [22]
(64)	(*trans*, *trans*)-Farnesol	Flowers [22]	(89)	α-Pinene	Flowers [22]
(65)	Linalool	Flowers [21]	(90)	β-Pinene	Flowers [22]
(66)	(*E*)-Nerolidol	Flowers [21]	(91)	Theaspirane A	Flowers [21]
(67)	Hexanal	Flowers [22]	(92)	2-Isobutyl-3-methoxypyrazine	Flowers [21]
(68)	Heptanal	Flowers [21]	(93)	Indole	Flowers [22]
(69)	Octanal	Flowers [21]	(94)	Benzyl alcohol	Flowers [22]
(70)	Nonanal	Flowers [22]	(95)	Phenethyl alcohol	Flowers [22]
(71)	Decanal	Flowers [22]	(96)	Benzaldehyde	Flowers [22]
(72)	Neral	Flowers [22]	(97)	Phenylacetaldehyde	Flowers [21]
(73)	Geranial	Flowers [22]	(98)	4-Methoxy benzaldehyde	Flowers [22]
(74)	Citronellal	Flowers [22]	(99)	Methyl benzoate	Flowers [22]
(75)	Farnesal	Flowers [22]	(100)	Methyl salicylate	Flowers [22]
(76)	6-Methyl hept-5-en-2-one	Flowers [22]	(101)	3-phenyl lactic acid	Leaves [29]
(77)	*cis*-β-Ocimene	Flowers [22]	(102)	Benzyl benzoate	Flowers [22]
(78)	β-Myrcene	Flowers [22]	(103)	Benzyl salicylate	Flowers [22]
(79)	*Allo*-ocimene	Flowers [22]	(104)	3-(3-indolyl) lactic acid	Leaves [29]
(80)	*trans*-β-Ocimene	Flowers [22]	(105)	Indole-3-lactic acid methyl ester	Leaves [29]
(81)	Terpinolene	Flowers [22]	(106)	Megastigmatrienone I	Flowers [21]
(82)	γ-Terpinene	Flowers [21]	(107)	Megastigmatrienone II	Flowers [21]
(83)	Terpinen-4-ol	Flowers [22]	(108)	Megastigmatrienone III	Flowers [21]
(84)	α-Terpineol	Flowers [22]	(109)	Megastigmatrienone IV	Flowers [21]

**Table 4:** Phenolic Compounds, Coumarin, and Flavonoids from *Brugmansia suaveolens*.

No.	Compound Name	Parts Used
(110)	Acanthoside B	Flowers [55]
(111)	Scopoletin 7-O-β-d-galactopyranoside	Flowers [55]
(112)	Kaempferol	Flowers [55]
(113)	Kaempferol 3-O-α-l-arabinopyranoside	Leaves [29]
(114)	kaempferol 3-O-α-l-arabinopyranosyl-7-O-β-d-glucopyranoside	Leaves [29]
(115)	Kaempferol 3-O-β-d-glucopyranosyl-(1′′′→2″)-O-α-l-arabinopyranoside	Leaves [56]
(116)	Kaempferol 3-O-β-d-glucopyranosyl-(1′′′→2″)-O-α-l-arabinopyranoside-7-O-β-d-glucopyranoside	Leaves [56]
(117)	Kaempferol 3-O-β-d-[6′′′-O-(E-caffeoyl)]-glucopyranosyl-(1′′′→2″)-O-α-l-arabinopyranoside-7-O-β-d-glucopyranoside	Leaves [56]
(118)	Kaempferol 3-O-β-d-[2′′′-O-(E-caffeoyl)]-glucopyranosyl-(1′′′→2″)-O-α-l-arabinopyranoside-7-O-β-d-glucopyranoside	Leaves [56]

**Table 5:** Steroids from *Brugmansia suaveolens*.

No.	Compound Name	Parts Used
(119)	20-hydroxyecdysone	Flowers [55]
(120)	Physalindicanol A	Leaves [29]
(121)	Physalindicanol B	Leaves [29]

**Table 6:** Hydrocarbons from *Brugmansia suaveolens*.

No.	Compound Name	Parts Used
(122)	Pentacosane	Flowers [21]
(123)	Heptacosane	Flowers [21]
(124)	Nonacosane	Flowers [21]
(125)	Hentriacontane	Flowers [21]
